# Small Molecule Compounds Inhibit Varicella-Zoster Virus Replication by Targeting the Portal Protein–Capsid Interface

**DOI:** 10.3390/v17111496

**Published:** 2025-11-12

**Authors:** Julius Svensmark, Emily Polk, Ellyn Kornfeind, Whitney Lane, Melissa A. Visalli, Robert J. Visalli

**Affiliations:** 1 Department of Biomedical Sciences, Mercer University School of Medicine, Savannah, GA 31404, USA; juliussvensmark@gmail.com (J.S.);; 2 Department of Biological Sciences, Purdue University-Fort Wayne, Fort Wayne, IN 46805, USA; wclane30@gmail.com; 3 Department of Biological Sciences, University of Alabama, Tuscaloosa, AL 35487, USA

**Keywords:** Varicella-zoster virus, antivirals, DNA encapsidation, portal protein

## Abstract

The Varicella-zoster virus (VZV) open reading frame 54 (ORF54) gene encodes an 87 kDa monomer that oligomerizes to form the pORF54 portal dodecamer. Located at a single viral capsid vertex, the portal facilitates the translocation of the newly synthesized viral genome into the preformed empty capsid. Previously described α-methylbenzyl thiourea compounds were shown to inhibit VZV DNA encapsidation, likely by targeting pORF54. In this study, drug resistant isolates were obtained via passage of VZV in increasing concentrations of one analog, Compound I (Comp I). Mutations identified in four compound resistant isolates (amino acids 48, 304, 324 and 407) all localized to a region of the portal that was predicted to interface with capsid proteins. The portal is known to undergo significant conformational changes at the portal–capsid interface during DNA encapsidation. A set of recombinant viruses was designed to reveal the chemical and physical importance of each of the resistance mutations at the portal–capsid interface, the proposed binding site of the compound series. In addition, we employed a novel complementing cell line to show that despite the presence of the portal in the virion, DNA encapsidation did not occur. We propose that a-methylbenzyl thiourea compounds perturb interactions at or near the portal–capsid interface and prevent conformational changes needed to support DNA encapsidation.

## 1. Introduction

Infection with the human alphaherpesvirus Varicella-zoster virus (VZV) is characterized by an initial lytic state where new virions are produced and spread via viremia, followed by establishment of a latent state in neurons of the sensory nerve ganglia including the dorsal root and cranial nerve ganglia [1,2]. Primary infection typically occurs in childhood and results in varicella (chickenpox) presenting as a rash with vesicular skin lesions. Although normally a self-limiting disease in children, primary infection in adults can result in more severe symptoms with secondary complications like viral pneumonia or fatal encephalitis. Reactivation of VZV, typically in the context of advancing age and/or a decline in VZV-specific cell-mediated immunity, can result in herpes zoster (shingles). Herpes zoster typically presents as a painful, unilateral vesicular rash following a dermatomal distribution. In some individuals, this is followed by the development of postherpetic neuralgia (PHN), which is characterized by persistent radicular burning pain that can continue for months or even years after resolution of the rash [3]. Beyond varicella and zoster, there are reports of an increased risk of stroke amongst children and adults following VZV infection [4]. Recent findings suggest that VZV may also contribute to giant cell arteritis, a condition leading to inflammation and occlusion of the arteries [4,5]. Immunocompromised individuals are at increased risk for both morbidity and mortality with primary infection and reactivation [6].

### 1.1. VZV Treatment Options

The introduction of a live attenuated varicella vaccine for immunization of healthy children and adults has effectively reduced morbidity and mortality worldwide [7,8,9]. More recently, a VZV glycoprotein E adjuvanted vaccine was introduced that reduces the incidence and severity of zoster in the elderly [10]. VZV vaccines have greatly reduced disease worldwide, but despite their relative success, there remains a critical need for therapeutic options [11,12,13,14].

The viral DNA polymerase is the primary target of currently approved antivirals for treating VZV infections. Drugs such as Acyclovir (ACV), Valaciclovir, Famciclovir, and Penciclovir are all nucleoside analogs that require phosphorylation by the VZV thymidine kinase (TK) for activation [2,11]. Subsequent phosphorylation by cellular kinases fully activates the compounds, resulting in the inhibition of viral DNA replication. Nucleoside analogs are the standard for treatment for both immunocompetent and immunosuppressed patients [2,15]. Acyclovir is effective at treating varicella when given early, preferably within 24–72 h of the rash appearing. It can reduce the number of lesions, shorten the duration of fever, and lessen the severity of symptoms [16]. Acyclovir can reduce the severity and frequency of zoster if treatment begins less than 72 h after onset of the rash, yet some patients still experience complications related to PHN after resolution [17]. In addition, there are multiple case reports showing clinical isolates of VZV with decreased susceptibility to ACV [18,19,20] mainly due to mutations in TK or DNA polymerase [21,22]. Brivudine, a potent nucleoside analog approved in some European countries for VZV treatment, also requires phosphorylation by TK and is ineffective for treating ACV resistant strains of VZV [23]. Along with brivudine, a closely related drug, sorivudine, was linked to deaths and the development of acute hepatitis when co-administered with the anticancer drug 5-fluorouracil [24,25,26]. ACV resistant VZV can have fatal consequences in immunocompromised patients [11]. The pyrophosphate analog foscarnet or nucleoside analog cidofovir can be used to treat ACV-resistant infections, although in some cases there may be severe side effects including nephrotoxicity [27].

There are several promising drugs at various stages of development [11]. Bicyclic pyrimidine nucleoside analogs (BCNA) and valomaciclovir are both nucleoside analogs that display high selectivity that effectively inhibits VZV replication [28,29]. Despite their promising profile, these drugs are TK-dependent and therefore would not provide an alternative for the treatment of ACV-resistant VZV. Acyclovir ProTide derivatives are nucleotide analogs that bypass the requirement for VZV TK and thereby retain activity against some ACV-resistant VZV [30]. 4-Oxo-dihydroquionolines show broad-spectrum activity against viral DNA polymerases in vitro, although no clinical trials have been reported [31]. A pyrazolo derivative (35B2) was shown to inhibit capsid assembly and thus may represent a novel VZV inhibitor of ACV-resistant strains [32]. No in vivo results have been reported.

Our laboratory focuses on α-methylbenzyl thiourea compounds as novel VZV DNA encapsidation inhibitors. These compounds have shown promising results in vitro [33] and in vivo [34]. Compounds from the series have excellent therapeutic properties, displaying low inhibitory concentrations (EC_50_ 0.03–1 μg/mL) and virtually no cellular cytotoxicity (CC_50_ > 15 μg/mL) [33,35]. The compounds were also active against acyclovir resistant VZV strains [33]. Cells infected with VZV in their presence accumulate empty viral capsids in the cell nucleus, and the absence of DNA filled capsids confirmed that this compound series inhibits viral DNA encapsidation [33]. An HCMV encapsidation inhibitor, letermovir [34] was approved to manage cytomegalovirus disease and thus provides proof of principle that inhibition of viral DNA encapsidation is a viable therapeutic approach for herpesviruses. We have shown that α-methylbenzyl thiourea compounds are more potent than acyclovir [33], have a novel mechanism of action [33], and are efficacious in vivo [34] and therefore may represent a promising treatment option.

### 1.2. DNA Encapsidation and the VZV Portal Protein (pORF54)

Viral scaffold proteins in the nucleus of an infected cell direct newly synthesized capsid proteins to assemble and form empty procapsids. Following assembly, the scaffold proteins are cleaved by a viral protease and released from the procapsid, resulting in a conformational transformation of the spherical procapsid into an icosahedral shaped B-type capsid. Concomitantly, the viral terminase associates with the capsid, using the portal protein as a docking site, and recognizes newly synthesized viral DNA in a sequence specific manner. ATPase activity provided by the terminase funnels the DNA through the portal, filling the capsid with a single unit-length viral genome, resulting in a dense core C-type capsid.

Specific for VZV, viral proteins essential for the DNA encapsidation process have been identified. These include the dodecameric portal protein pORF54, the trimeric viral terminase pORF30-pORF42/45-pORF25, and accessory proteins pORF34, pORF26, and pORF43 [36,37,38,39,40,41,42,43,44,45,46,47,48]. Deletion of any of these genes from the viral genome results in accumulation of empty capsids in the nucleus [49,50]. Thus, viral DNA encapsidation proteins are potential targets for antiviral therapy.

Electron microscopy studies of purified HSV-1 pUL6 [51], HCMV pUL104 [52,53], VZV pORF54 [54,55], and EBV BBRF1 [56] provide valuable insight to the structural nature of herpesvirus portals. In all cases portal protein monomers form a dodecameric structure, embedded at a 5-fold vertex of the capsid, forming a channel for the translocation of viral DNA. Structural models characterizing the portal at the capsid vertex have been described for HSV-1 [57,58,59,60], VZV [61], HCMV [62,63], EBV [64,65], and HHV-8 [66].

Previous studies have identified a series of α-methylbenzyl thiourea compounds as novel VZV encapsidation inhibitors [33,67]. These compounds merit study as potential drug candidates [34] and also as tools to dissect the process of DNA encapsidation.

The α-methylbenzyl thiourea compounds are thought to disrupt the encapsidation process by possibly preventing (i) portal oligomerization, (ii) association with the capsid vertex, (iii) association with one or more terminase subunits, and/or (iv) conformational changes to the capsid during the translocation of viral DNA into the procapsid. Understanding the mechanism of action is essential to further development of the thiourea compounds as VZV inhibitors.

## 2. Materials and Methods

### 2.1. Bacterial Artificial Chromosomes, Plasmids, Bacterial Strains, Baculoviruses, and Oligonucleotide Primers

A comprehensive list describing these reagents is provided in Table 1: Reagents and materials used in this study.

### 2.2. Cells and Viruses

ARPE19 cells (human retinal pigmented epithelial cells; ATCC CRL-2302) were maintained at 37 °C and 5% CO_2_ in minimal essential media (MEM) supplemented with 5% fetal bovine serum (FBS), 2 mM L-glutamine, 100 U/mL penicillin, 100 μg/mL streptomycin, and 0.25 μg/mL amphotericin B.

ARPE19 cells were used to generate virus stocks after infection with VZV strain Ellen [33], or transfection of the original VZV_LUC_ [68] or ORF54 recombinant BAC DNAs. Infected cell monolayers were dislodged with trypsin, suspended in 90% FBS and 10% dimethyl sulfoxide, and frozen at −80 °C overnight. Cell associated stocks were moved to liquid nitrogen for storage.

Isolation of VZV strain Ellen Comp I-resistant isolates 1rA and 1rB was described previously [33]. Two additional isolates, 1rC and 3rA, were generated using the same procedure. Cells were infected with cell-associated VZV Ellen at an MOI of ∼0.01 in the presence of 0.26 μg/mL Comp I. After >50% cytopathic effect (CPE) was observed, an infected cell stock was prepared, and a portion was used to infect fresh cell monolayers in the presence of two-fold increasing concentrations of Comp I. The process was repeated four times, and the continued observation of CPE upon passage indicated that virus was able to replicate in the presence of Comp I. In total, four Comp I-resistant viruses were derived from separate resistant virus pools, plaque purified, and cell-associated stocks stored in liquid nitrogen. The isolation of resistant mutants was challenging. Most attempts to generate resistant isolates by passaging virus-infected cells in increasing concentrations of Comp I were not always successful. Fewer than 1% of the independently passaged pools contained resistant viruses.

A previously described ACV-resistant VZV strain Ellen isolate (Ellen-ACVrA) was isolated as described above, except that the virus was passaged in increasing concentrations of acyclovir [33].

A derivative of the previously described ARPE54C50 cell line [69] expressing a truncated version of pORF54 was generated to include a point mutation conferring resistance to Comp I. The codon-optimized ORF54 gene (GenScript USA Inc., Piscataway, NJ, USA), truncated to the first 2160 of 2310 base pairs and lacking the final 50 amino acids, was previously cloned downstream of the EF-1α promoter in a lentiviral expression vector (VectorBuilder Inc., Chicago, IL, USA) containing a puromycin resistance cassette [69]. To generate the resistant variant, this construct was modified to incorporate the Y324C substitution by site-directed mutagenesis. Lentivirus was produced and used to transduce ARPE19 cells, followed by puromycin selection to establish a stable cell line, designated ARPE Y324C. This cell line expresses an endogenously truncated pORF54 protein containing the Y324C mutation. Western blot analysis confirmed expression of the 82 kDa truncated pORF54 variant (Figure 6, lane 1). The ARPE Y324C line was used to investigate the effects of Comp I on the incorporation into virions and proteolytic processing of virally derived pORF54 HA (Section 3.5).

### 2.3. DNA Mutagenesis

ORF54 mutations were generated using a site-directed mutagenesis kit (Thermo Fisher Scientific, Waltham, MA, USA), specific mutagenic primers, and plasmid pJET54 (Table 1) as the template [69]. Primers incorporating each individual mutation were synthesized by IDT (Coralville, IA, USA) and are listed in Table 1. PCR products of the correct size were purified from agarose gels, ligated using T4 DNA ligase (Thermo Fisher Scientific, Waltham, MA, USA) and transformed into *E. coli.* Colonies were incubated in 5 mL LB with ampicillin at 37 °C overnight in a shaking incubator. Plasmid DNA was purified using the Thermo Scientific GeneJet kit (Thermo Fisher Scientific, Waltham, MA, USA) and digested with restriction enzyme BglII to confirm the presence of ORF54. Plasmids were sequenced to confirm the correct nucleotide substitution(s).

### 2.4. DNA Sequencing

DNA sequencing of pJet and VZV BAC DNAs was performed by Eurofins MWG Operon (Louisville, KY, USA) to confirm the presence of the desired mutation.

### 2.5. Agarose Gel Electrophoresis and DNA Restriction

PCR products and DNA digests were analyzed on 0.8% agarose gels stained with ethidium bromide (EtBr) and visualized using a BioRad Universal Hood Gel Doc System.

### 2.6. Recombineering ORF54 Mutants

ORF54 DNA containing the desired point mutations were incorporated into the VZV genome by recombineering [70]. Previously described VZV_LUC_ BACs were used as the recipient vectors for generating amino acid 304, 324, and 407 mutants (BAC D54S) and amino acid 48 mutants (BAC D54L) [50]. A 5 mL LB-chloramphenicol (25 μg/mL) culture of SW102 cells containing BAC Δ54S or Δ54L was grown overnight at 32 °C and diluted 1:50 in 25 mL LB-chloramphenicol (25 μg/mL) in a 100 mL Erlenmeyer flask. Cells were incubated at 32 °C with shaking to an optical density of 0.4 (OD_600_). Cells were heat-shocked at 42 °C in a shaking water bath for 15 min then cooled in an ice water bath for 2 min. Cells were transferred to a cool 50 mL centrifuge tube and pelleted by centrifugation at 4500 rcf at 4 °C for 5 min. The supernatant was removed, and the cell pellet was resuspended in 25 mL ice-cold, sterile double distilled water (ddH_2_O) by gently swirling the mixture in an ice water bath. After repeating the washing step, cells were pelleted and resuspended in 1 mL ice-cold ddH_2_O. Resuspended bacteria were transferred to a 1.5 mL microfuge tube and pelleted at 13,200 rpm 4 °C for 1 min. The supernatant was removed, and cells were resuspended in 75 μL ice-cold ddH_2_O. A volume of 30 μL of bacterial resuspension was mixed with 500 ng of PCR-amplified VZV ORF54 DNA containing the desired mutation as described below.

For recombineering of all mutations, primers MU23/MU31, specific for the full length ORF54 gene including regions of flanking genes ORF53 and ORF55, were used to create PCR products. The DNA amplicon/cell solution was electroporated in a 0.1 cm cuvette using a Bio-Rad Gene Pulser X Cell (Bio-Rad Laboratories, Hercules, CA, USA). After electroporation, cells were transferred to a 50 mL baffled conical flask with 10 mL LB and allowed to recover in a 32 °C shaker for 4 h. One mL of recovery culture was transferred to a 1.5 mL microfuge tube and pelleted in a centrifuge for 1 min at 13,200 rpm at room temperature. The supernatant was removed and the pellet washed in 1 mL M9 buffer four times. Upon the last resuspension, 100 μL of the cell solution was plated on a M63 minimal media plate containing 2-deoxygalactose (DOG) to negatively select cells still harboring the *galK* gene. Plates were incubated at 32 °C for 3–5 d. Individual colonies were screened for successful recombination by preparing an overnight culture of 5 mL LB-chloramphenicol containing a single colony picked from the DOG plate. Cells were pelleted by centrifugation at 4500 rpm at room temperature for 5 min. BAC DNA was isolated from pelleted cells using the NucleoBond BAC 100 kit (Clontech Laboratories, Mountain View, CA, USA). The DNA was subsequently screened by generating an ORF54 amplicon with primers MU23/31. PCR products were then digested with restriction enzyme XhoI (there is a diagnostic XhoI site at ORF54 bp 1372) and analyzed by agarose gel electrophoresis. All positive recombinant colonies were confirmed to contain the desired mutation by DNA sequencing.

### 2.7. BAC DNA Isolation and Transfection of ARPE19 Cells

BAC DNAs were prepared using a NucleoBond Xtra BAC purification kit (Macherey-Nagel GmbH & Co. KG, Düren, Germany). Cells containing recombinant BACs were grown overnight in 400 mL LB with 25 mg/mL chloramphenicol. Cells were pelleted at 4500 rcf at 4 °C for 10 min and resuspended in 60 mL RES-BAC buffer. Cells were lysed by adding 60 mL of LYS-BAC buffer and incubating for 5 min at room temperature. Sixty milliliters of NEU-BAC buffer was added, and the lysate was incubated for 10 min on ice. The precipitate was pelleted at 4500 rcf for 10 min and the supernatant was applied to a NucleoBond column. DNA was washed with 30 mL WASH-BAC buffer and eluted by adding 15 mL of ELU-BAC buffer pre-warmed to 70 °C. Isopropanol was added to precipitate the DNA. DNA was pelleted by centrifuging at 15,000 rcf at 4 °C for 30 min then washed once in 70% EtOH, air dried, and resuspended in 75 μL 10 mM Tris-HCL, pH 8.5.

Five micrograms of recombinant BAC DNA were used to transfect ~1 × 10^6^ ARPE19 cells. DNA was diluted in 2 mL MEM and mixed with 8 μL of PLUS Reagent and 20 μL Lipofectamine (ThermoFisher Scientific, Waltham, MA, USA). This mixture was incubated for 15 min at room temperature and added to a 100 mm dish containing ARPE19 cells in MEM with 2% FBS. Cells were incubated for 2–3 d and observed for GFP-positive plaques.

### 2.8. Replication Kinetics

Confluent twelve well plates containing ~3 × 10^5^ cells in MEM and 2% FBS were infected in triplicate with 300 PFU/well of each mutant virus and VZV_LUC_. Infected monolayers were incubated at 37 °C for 6, 24, 48, 72, 96, and 120 h, then harvested by washing with PBS, adding 1× luciferase cell lysis buffer for 15 min at room temperature and freezing at −80 °C. Replication was determined via relative luminescence units (RLUs) using a firefly luciferase glow assay kit (Thermo Fisher Scientific, Waltham, MA, USA) and measured on a Veritas microplate luminometer (Promega Corporation (Turner BioSystems), Madison, WI, USA). The data were graphed as the averages ± standard errors for triplicate samples.

### 2.9. Yield Reduction Assay (For Recombinant VZV_LUC_ and TN Insertion Viruses)

Virus-infected ARPE19 cells were exposed to increasing concentrations of α-methylbenzyl thiourea analog Comp I to determine their sensitivity. ARPE19 cells (~3.8 × 10^5^/well) were infected with 300 PFU (infectious centers) of VZV-infected cell stock per well (MOI ~0.0008). The sensitivity of mutants was determined by diluting the compound to concentrations (0.03, 0.06, 0.125, 0.250, 0.500, 1.00, or 2.00 μg/mL) in 2% MEM containing 0.3% dimethyl sulfoxide (DMSO). Positive-control wells were VZV-infected cells in MEM containing 0.3% DMSO without the compound. Monolayers were incubated for 5 d at 37 °C, washed with PBS, and harvested in 1x luciferase cell lysis buffer. Relative luminescence units (RLU) were determined for each sample using a firefly luciferase glow assay kit (Thermo Fisher Scientific, Waltham, MA, USA. The data were graphed as the averages from triplicate samples ± standard errors.

### 2.10. ELISA and Plaque Reduction Assays (For Ellen Resistant Isolates)

The plaque reduction assay (PRA) and VZV high-throughput robotic ELISA were described in detail previously [33] and will only be briefly summarized below.

For the PRA, ARPE19 cell monolayers in 60 mm tissue culture dishes (3.5 × 10^6^ cells/dish) were infected in triplicate with approximately 50 to 100 PFU of VZV Ellen or compound-resistant VZV Ellen virus-infected cell stock. Sensitivity of mutants was determined by incubating infected cells in increasing concentrations of Comp I (0.05, 0.5, or 2 μg/mL) in 2% MEM containing 0.3% DMSO. Monolayers were incubated for 6 d at 37 °C, fixed with 10% formalin, and stained with crystal violet. Plaques were counted and the data presented as the mean of three dishes for each concentration.

An automated enzyme-linked immunosorbent assay (ELISA) was used to measure the inhibition of VZV replication by Comp I. A primary monoclonal VZV glycoprotein B (gB) antibody (Advanced Biotechnologies, Inc., Columbia, MD, USA) was used to detect the relative amount of glycoprotein B expressed in VZV Ellen compared to compound-resistant VZV Ellen in the presence or absence of compound.

### 2.11. SDS PAGE, Western Blotting, and Antibodies

Cell extracts or purified virions were solubilized in 2× Laemmli sample buffer and separated via SDS-PAGE on 12-well 10% Criterion gels (BioRad Laboratories, Hercules, CA, USA). Proteins were transferred to PVDF membranes for immunoblot analysis. pORF54 and major capsid protein (mcp; ORF40) were detected using monoclonal antibodies VZ 54.08 and VZ 40.1.03, respectively [Center for Proteomics University of Rijeka, Rijeka, Croatia [71]. pORF54 tagged with the HA epitope was detected using anti-HA antibody clone 2–2.2.14 (Thermo Fisher Scientific, Waltham, MA, USA). Primary antibodies were detected using an HRP-conjugated goat anti-mouse secondary antibody (Thermo Fisher Scientific (Invitrogen), Waltham, MA, USA). Chemiluminescent detection was performed using the SuperSignal West Pico chemiluminescent substrate system (Thermo Fisher Scientific, Waltham, MA, USA) and proteins were visualized after exposing the membrane to autoradiography film (Santa Cruz Biotechnology, Inc., Dallas, TX, USA) or captured digitally on an Azure 600 imaging system. Some blots were probed multiple times after stripping with 6 M guanidine hydrochloride (GnHCL) [72].

### 2.12. Purification of VZV Virions

Gradient purified virions were isolated as described previously with minor modifications [69]. Ten 100 mm culture dishes of ARPE 19 or Y324C cells were infected with cell-associated VZV stocks in the presence or absence of Comp I. Cells were harvested once cytopathic effect (CPE) exceeded 80%. Infected cells were rinsed twice with phosphate-buffered saline (PBS), scraped into 2 mL of minimal essential medium (MEM), and collected by centrifugation at 1500 rpm for 5 min. The pellet was resuspended in 3 mL of MEM and disrupted by 20 strokes with a 40 mL Tenbroeck tissue homogenizer (Corning). Cell debris was removed by low-speed centrifugation in a 15 mL conical tube at 1000 rpm for 5 min. The clarified supernatant was carefully layered onto a 20–60% (*w*/*v*) sucrose gradient prepared in PBS and centrifuged for 1 h at 24,300 rpm (approximately 1.0 × 10^5^× *g*) in an SW41 swinging-bucket rotor. The virion-containing fraction, visible as an opalescent band, was collected using a Pasteur pipette, adjusted to 28% sucrose in PBS, and centrifuged for 1 h at 21,000 rpm (7.5 × 10^4^× *g*) using the same rotor. The resulting virion pellet was resuspended in PBS using a wide-bore pipette tip and incubated overnight at 4 °C to complete solubilization.

## 3. Results

### 3.1. Isolation of an Original Panel of VZV Drug Resistant Isolates

**Figure 1:**
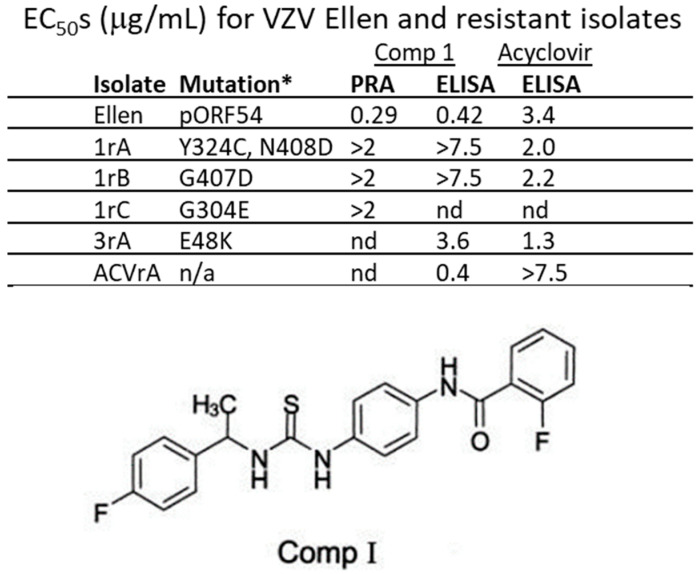
EC_50_s for VZV Ellen and resistant isolates. The initial resistant isolates were all derived from repeated passage of VZV strain Ellen-infected cells in the presence of increasing concentrations of Comp I. Mutations conferring resistance were mapped to pORF54. The amino acid changes and residue numbers are indicated for each isolate. All resistant isolates had EC_50_s ~5–20-fold greater than parental VZV Ellen. * Amino acid change in pORF54; n/a—not applicable; nd—not done; 2 μg/mL—maximum concentration of drug tested in plaque reduction assay (PRA); 7.5 μg/mL—maximum concentration of drug tested in ELISA; Comp I—2-Fluoro-N-(4-(3-[1-(4-fluoro-phenyl)-ethyl]-thioureido)-phenyl)-benzamide (structure shown) [33,35].

Previous studies identified and confirmed via marker rescue two VZV strain Ellen a-methyl benzyl Comp I resistant isolates with mutations that mapped to ORF54 amino acids 324/408 and 407 [33]. Two additional drug-resistant isolates were generated by passaging VZV strain Ellen-infected ARPE19 cells in the presence of increasing concentrations of Comp I. Sequence analysis of the ORF54 gene identified mutations that potentially conferred resistance. Isolates were compared to the parent strain, VZV Ellen, or an acyclovir resistant isolate, ACVrA (Figure 1). EC_50_ values were >2.0 μg/mL for the resistant isolates compared to 0.29–0.42 μg/mL for VZV Ellen. An acyclovir resistant isolate (ACVrA) was sensitive to Comp I (0.4 μg/mL). Resistant isolates were ~7–18-fold more resistant to Comp I compared to parent virus.

**Figure 2:**
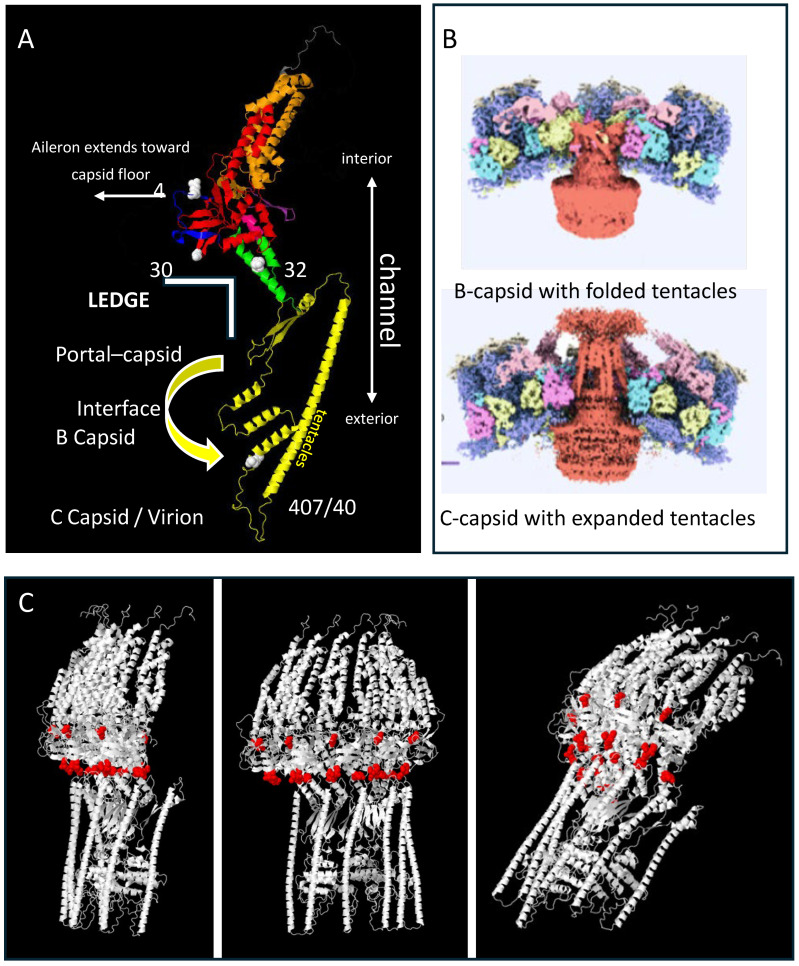
**Alpha-fold model for the VZV portal monomer and multimer**. The full-length pORF54 portal protein sequence, comprising 769 amino acids, was submitted to the AlphaFold Protein Structure Database server for structural prediction [73]. The amino acid sequence was provided in FASTA format and processed using the AlphaFold default settings to generate a high-confidence three-dimensional structural model. In panel (**A**), the domains are color coded on the model as follows: Blue N-terminus (aileron?) (1–69, only 41–69 shown), Red Wing (70–99 + 187–311), Orange Wall (100–186 + 586–650), Green Stem (312–349 + 544–557), Yellow Clip (including turret) (341–543), Purple b-hairpin (tunnel loop) (568–585), and Gray C-term extended crown (651–769, only 651–668 shown). Features and notes: (i.) The clip / turret region has not been completely resolved for any herpesvirus portal monomer. However, these domains are predicted to collapse or fold closer to the portal core in the empty B-capsid. In the DNA filled capsid, the clip and turret regions (which include amino acids 407/408) extend outward beyond the capsid floor (panel (**B**); insets adapted with permission from Cao L. et al., 2024 [61]). (ii.) The region noted as the portal “ledge” interfaces with the capsid floor and may interact with one or more capsid proteins depending on its conformation. Mutations that conferred resistance (48, 304, 324, and 407) are predicted to be part of the portal “ledge” and noted by the white space-filled atoms. (iii.) The model could not accurately predict the aileron region that includes amino acid 48, but this region likely extends out toward the capsid interface as has been shown for other herpesvirus portals [62]. Panel (**C**) represents the multimer prediction of the monomer noted in panel A. Half of a complete portal (6 monomers) is shown at different angles. The red space-filled atoms represent the location of resistance loci including g48, 304, 304–306 loop, and 324. These cluster around the portal equator, the region predicted to contact capsid proteins.

Although the complete structure of pORF54 and other herpesvirus portals is still unresolved, recent studies have revealed structurally conserved regions that help approximate the locations of mutations responsible for resistance to Comp I. The mutations appeared to cluster near the intersection of the portal ledge (made up of the lower wing-stem-clip/turret domains) and the viral capsid (Figure 2). We aimed to further characterize resistance mutations at the portal–capsid interface to more precisely define the mechanism of action of the thiourea series.

### 3.2. Targeted Mutagenesis of ORF54

Recombineering using previously described VZV bacterial artificial chromosomes (BACs; [50,68]) were employed to generate the original, as well as conservative and non-conservative mutations at amino acids 48 (aileron or lower wing), 304 (lower wing), 324 (stem), and 407 (clip/turret). Each mutant was tested for its replication phenotype and resistance profile to one or more of the thiourea compounds. The nature of the amino acid substitution along with the observed resistance phenotype was used to further characterize their contribution to a potential compound binding site and for their relevance in potential interactions between the portal and viral capsid.

Targeted mutagenesis to generate single amino acids substitutions at pORF54 residues 48, 304, 324, or 407 was performed using paired oligonucleotides (Table 1) and VZV ORF54 plasmid template. ORF54 DNA amplicons containing the desired individual mutations were recombineered [70]. The ORF54 gene was sequenced for each recombinant BAC to confirm the presence of the desired mutation. All BAC constructs were digested with Sal I and compared to the parental VZV_LUC_ genome via agarose gel electrophoresis and EtBr staining. All BAC digests displayed a pattern identical to VZV_LUC_, confirming no gross alterations or rearrangements had occurred to the genome.

BAC DNAs containing point mutations were transfected into ARPE-19 cells. Infected cells were passaged to generate viral stocks that were subsequently titered on ARPE-19 monolayers. Infected-cell stocks were used to perform replication kinetics and plaque morphology analyses. All viruses contained the luciferase and GFP genes as previously described [68].

### 3.3. Replication Kinetics of Mutant Viruses

**Figure 3:**
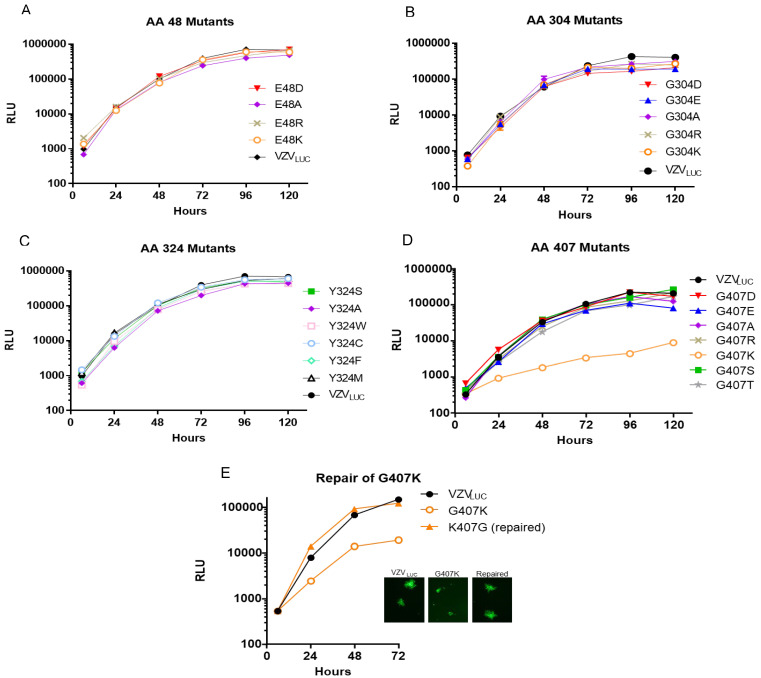
**Replication kinetics of VZV_LUC_, 48, 304, 324, and 407 recombinants.** ARPE-19 cell monolayers in 12-well plates were infected with ~300 PFU of cell-associated VZV_LUC_ or each recombinant virus. Three wells per time point were harvested in luciferase lysis buffer and viral replication was measured by firefly luciferase activity shown as relative luminescent units (RLU). Error bars representing the standard error of the mean (SEM) were consistently small and not visible on the log scale, underscoring the high reliability and reproducibility of the luciferase-based replication assay. Viruses were grouped by their resistance locus: (**A**) amino acid 48 mutants, (**B**) amino acid 304 mutants, (**C**) amino acid 324 mutants, and (**D**) amino acids 407 mutants. Panel (**E**) compares the replication phenotype of the crippled G407K recombinant to VZV_LUC_ and a repaired virus (K407G). The inset shows representative plaque phenotypes for the three viruses.

**Replication of N-terminal/aileron mutants (amino acid 48):** Replication kinetics for “48” mutants are shown in Figure 3A. All mutants showed similar growth kinetics to each other and when compared to VZV_LUC_. The data suggests that substitutions at residue 48 did not disrupt portal structure or function.

**Replication of Lower Wing (amino acid 304) and Stem (amino acid 324) mutants**: Figure 3B,C show the replication kinetics for all “304” and “324” mutants. All viruses replicated with approximately the same kinetics. The data suggests that substitutions at residues 304 or 324 did not disrupt portal structure or function.

**Replication of Portal Clip/Turret mutants (amino acid 407):** Figure 3D,E show the replication kinetics for all “407” mutants. Only the 407 lysine (K) mutant was significantly impaired for replication. The replication kinetics of a 407K repaired virus was similar to VZV_LUC_ (Figure 3E). The data suggests that substitutions at residue 407 did not disrupt portal structure or function save for the positively charged lysine substitution. 407K plaques were significantly smaller than VZV_LUC_ (Figure 3E). The other seven mutants had 72 h plaque morphologies indistinguishable from VZV_LUC_.

### 3.4. Activity of an α-Methylbenzyl Thiourea Compound (Comp I) Against VZV Mutants

**Figure 4:**
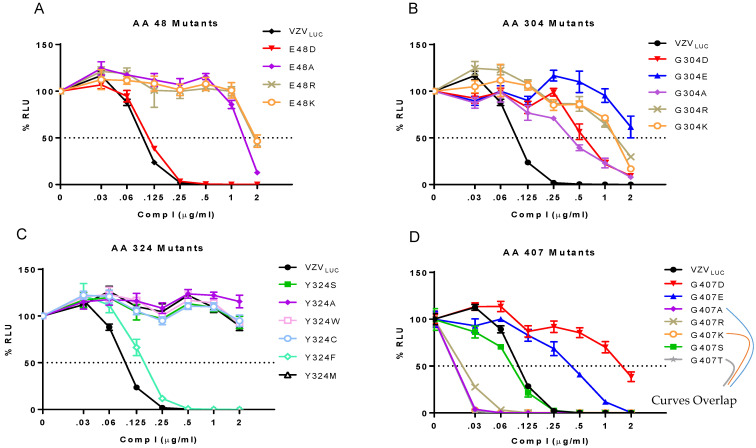
**Yield reduction assays for VZV_LUC_, 48, 304, 324, and 407 recombinants.** ARPE-19 cell monolayers in 12-well plates were infected with ~300 pfu of cell-associated VZV_LUC_ or each recombinant virus and incubated with increasing concentrations of Comp I. After 5 d, three wells per drug concentration were harvested in luciferase lysis buffer and analyzed for firefly luciferase activity. Each point represents the average of three wells (± SEM) converted to % relative luminescent units (% RLU) compared to a value of 100% corresponding to each virus in the absence of Comp I (0). Viruses were grouped by their resistance locus: (**A**) amino acid 48 mutants, (**B**) amino acid 304 mutants, (**C**) amino acid 324 mutants, and (**D**) amino acids 407 mutants.

We hypothesized that mutant viruses with only the most conservative substitutions would retain sensitivity to the compound. The resistance phenotype of each mutant was examined in the presence of Comp I. Yield reduction assays with increasing concentrations of Comp I were performed to determine the resistance phenotype for each mutant at the four different loci (Figure 4, Table 2).

**Phenotype of N-terminal/aileron mutants (amino acid 48) to Comp I:** (Figure 4A) The original mutation at amino acid 48 was a non-conservative substitution going from a negatively charged glutamic acid to a positive charge lysine (E48K). Three additional mutants were engineered to determine if the negative charge at this locus was an important determinant of compound efficacy. Glutamic acid to either arginine (E48R) or alanine (E48A) were non-conservative substitutions that yielded resistant viruses. Only the conservative substitution to aspartic acid (E48D) that retained a negative charge resulted in a sensitive phenotype (EC_50_ 0.11 μg/mL) suggesting that a negatively charged amino acid is required to maintain compound efficacy.

**Figure 5:**
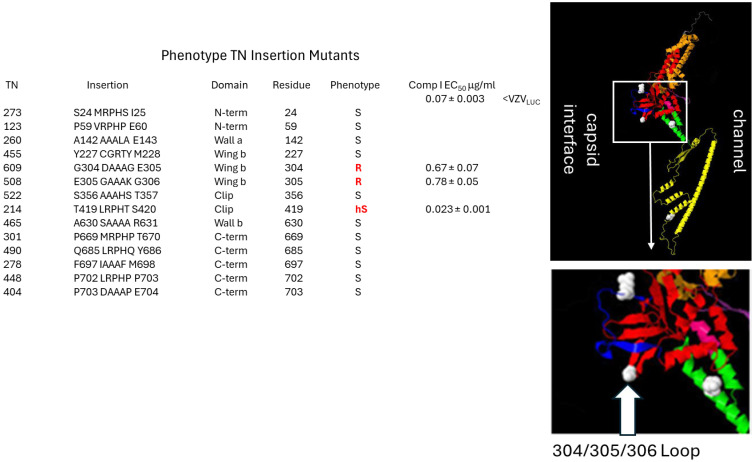
**Insertion mutants in the “ledge” proximal 304–306 wing loop confer resistance to Comp I.** A previously described pORF54 in-frame insertion library was screened for sensitivity to Comp I. The first column (TN) is the assigned recombinant number from the previous publication (TN library; [69]). The insertion column shows where a five amino acid in-frame insertion was identified for each TN. The domain column represents where the insertion mapped in the pORF54 monomer. The residue column indicates the last amino acid residue before the insertion. The phenotype column represents whether the TN recombinant was sensitive (S), hypersensitive (hS), or resistant (R) to Comp I compared to VZVLUC. EC_50_ values are provided for any resistant or hypersensitive recombinants. Resistance to Comp I was mapped to the lower portion of the portal wing, at a Glycine–Glutamic Acid–Glycine loop formed by amino acids 304–306. This loop appears to extend into the proposed portal ledge that interfaces with the capsid. Disruption or extension of this loop likely prevents compounds from binding efficiently (EC_50_ 0.07 for VZV_LUC_ versus EC_50s_ of 0.67 and 0.78 for TNs 609 and 508, respectively). An in-frame insertion at amino acid 419, in the clip/turret region, conferred hypersensitivity. We hypothesize that in the presence of Comp I, this mutation impedes a required conformational change in portal during DNA packaging.

**Phenotype of Lower Wing (amino acid 304) and Stem (amino acid 324) mutants to Comp I:** (Figure 4B,C) The original mutation at amino acid 304 was a non-conservative substitution of glycine, a non-polar, uncharged amino acid, to aspartic acid, a polar, negatively charged residue (G304E). Three additional mutants were engineered. Glycine to either arginine (G304R) or lysine (G304K) were non-conservative charged, polar substitutions that all yielded resistant viruses. These and the original mutation were all significantly bulkier than the wild-type glycine. Even a highly conservative small, non-polar substitution of glycine to alanine (G304A) resulted in a 4-fold increase in the EC_50_ (Table 2). Congruent with this finding were the phenotypes of two TN mutants containing random five amino acid in-frame insertions between pORF54 amino acids 304/305 (TN609) or 305/306 (TN508). Both were resistant (EC_50_s of 0.67 and 0.78 μg/mL, respectively) to Comp I (Figure 5). Based on the predicted structure of the portal lower wing, glycine 304 is part of a loop that extends into the portal ledge (Figure 5). Insertions into this loop may disrupt a putative compound binding pocket.

The original mutation at amino acid 324 was a non-conservative substitution of a tyrosine, an aromatic amino acid, to a cysteine (Y324C). Other than the tyrosine to phenylalanine (Y324F) substitution, all other amino acid substitutions tested at this position conferred resistance to Comp I (EC_50_ > 2 μg/mL). The Y324F substitution was sensitive with an EC_50_ similar to VZV_LUC_. Phenylalanine and the wild-type tyrosine are very similar in size and structure, differing only by the addition of an -OH group at the fourth position in the benzene ring. The substitution of tyrosine with tryptophan (Y324W), a larger aromatic amino acid, conferred resistance. All three of the non-aromatic substitutions (Y324S, Y324A, Y324M) displayed a resistant phenotype. It appears that there is a strong requirement for an aromatic substituent at the 324 locus and that size (bulk) is also a factor.

**Phenotype of Portal Clip/Turret mutant (amino acid 407) to Comp I:** (Figure 4D) Substitutions at amino acid 407 yielded a more complex phenotype than those observed at the other positions (Table 2). The original resistance mutation at amino acid 407 was a non-conservative substitution of glycine, a non-polar, uncharged amino acid, to aspartic acid, a polar, negatively charged amino acid (G407E). The substitution of glutamic acid (also a polar, negatively charged residue) yielded a similar resistance phenotype. All other substitutions, ranging from conservative alanine (G407A), threonine (G407T), and serine (G407S) to non-conservative arginine (G407R) and lysine (G407K), yielded a sensitive or hypersensitive phenotype (EC_50_s < VZV_LUC_). The portal clip/turret region encompassing amino acid 407 is different than the other resistance loci. The portal clip/turret is predicted to expand or extend from a collapsed state in the B-capsid to an extended state in the C-capsid (see Figure 7) upon DNA packaging [61,62,63]. Transitioning from an empty to a full capsid requires a series of conformational changes mediated by complex protein–protein interactions. The nature of the amino acid residue at 407 likely influences the ability of the portal to go through one or more required conformational changes during packaging. Interestingly, out of all the mutants in this study, the only one that had a replication defect was G407K (Figure 3E), further indicating the importance of this region in portal restructuring during genome packaging.

### 3.5. Dissecting the Mechanism of Action for Comp I

We propose that the two most likely possibilities for how a-methylbenzyl thiourea compounds disrupt DNA encapsidation are by (i) preventing portal from being incorporated into capsids or (ii) interfering with portal–capsid interactions during DNA encapsidation. We leveraged a unique aspect of VZV portal protein processing that can distinguish between pre- and post- DNA encapsidation, allowing us to more precisely investigate the mechanism of action for Comp I.

**Figure 6:**
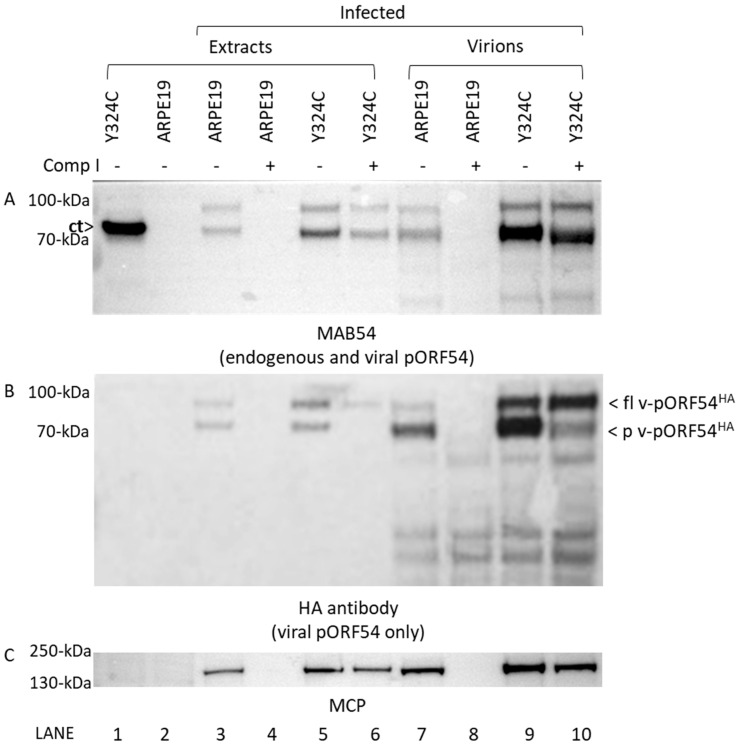
**Portal proteins are incorporated into the capsid but not processed in the presence of Comp I.** ARPE19 or Y324C cells were either uninfected or infected with VZV_LUC_54^HA^ in the absence (-) or presence (+; 4 μg/mL) of Comp I. Cells were harvested as total cell extracts (lanes 1–6) or for the isolation of sucrose gradient purified virions (lanes 7–10). Western blot was performed on all samples with (**A**) MAB54 to detect endogenous pORF54 (C-terminal truncated) and virally expressed pORF54^HA^, (**B**) HA antibody to detect only virally expressed pORF54^HA^, and (**C**) antibody to major capsid protein (MCP). Note (i) pORF54 expressed in Y324C cells is not detected using the HA antibody (Panel (**B**), lane 1) and (ii) the quantity of processed pORF54^HA^ is significantly reduced in Y324C virions from drug-treated cells (Panel (**B**), lane 10) versus untreated cells (Panel (**B**), lane 9). Key: [**ct**]—endogenously expressed c-terminally truncated pORF54 with a Y > C mutation at amino acid 324; [fl v-pORF54^HA^]—full length 87 kDa pORF54 containing a N-terminal HA epitope tag; [p v-pORF54^HA^]—post-DNA encapsidation processed 82 kDa pORF54 containing a N-terminal HA epitope tag.

As we showed previously, full length 87 kDa pORF54 is processed at the C-terminus during DNA encapsidation to yield an 82 kDa product that is the predominant form found in virions [69]. Although the significance of this cleavage event is unknown (i.e., it is not required for viral replication or DNA encapsidation), it is a useful marker for monitoring completion of DNA packaging. In combination with a cell line expressing endogenous truncated pORF54 (Figure 6A; ct) containing one of the mutations conferring resistance to Comp I (ARPE Y324C cells), we used this marker to study the effects of Comp I on the proteolytic processing of virally derived pORF54.

ARPE19 or Y324C cells were either uninfected or infected with VZV_LUC_54^HA^ in the presence or absence of Comp I. Cells were harvested and processed to yield total cell extracts or gradient purified virions. Western analysis showed that major capsid protein was present in all infected samples in the absence of compound (Figure 6C, lanes 3, 5, 7, and 9) while Y324C cells were required to support viral replication in the presence of Comp I (Figure 6C, compare lanes 4 and 6, and 8 and 10). Two additional Western blots were performed using (i) a monoclonal antibody, MAB54, that detected both endogenous truncated (ct) and viral pORF54 (Figure 6A) and (ii) an antibody to the HA epitope tag (Figure 6B) that detected only pORF54 (pORF54^HA^) expressed from the virus [69].

As expected, in the absence of Comp I, both full length (87 kDa) and processed (82 kDa) pORF54 were observed in infected cell extracts (Figure 6A, lanes 3 and 5) and purified virions (Figure 6, lanes 7 and 9). Notably, virally expressed pORF54^HA^ in virions purified from either ARPE19 (Figure 6B, lane 7) and Y324C (Figure 6B, lane 9) cells contained an abundance of processed pORF54 (p v-pORF54^HA^), suggesting that DNA encapsidation proceeded as usual.

**Figure 7:**
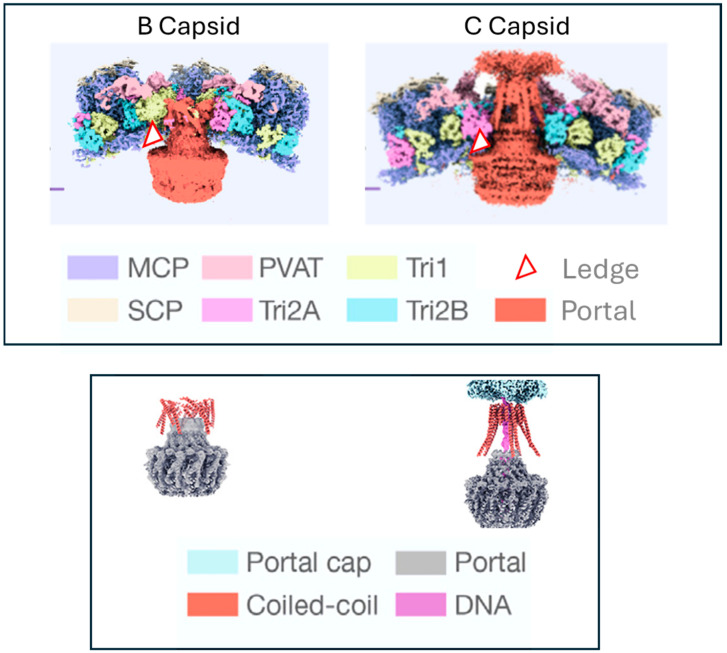
**Interactions at the portal—capsid interface.** Structure of the VZV portal in B and C capsids (adapted with permission from Cao L. et al. [61]). The top panel shows the portal in the context of the capsid proteins. The bottom panel shows the portal with the capsid proteins stripped away. The portal clip/tentacle region is thought to be folded down toward the portal stem in the B capsid. Upon DNA packaging the coiled-coil regions of the portal extend outward through the capsid protein layers where they eventually interact with the portal cap. The portal in its entirety moves outward and the ledge moves into proximity with one or more of the capsid proteins. The red and white triangle (top panel) depicts the location of the “portal ledge” at the interface of the portal oligomer and the capsid floor. The triangle represents the projected location of the VZV pORF54 resistance mutations at amino acids 48, 304, and 324. Key: MCP—major capsid protein; PVAT—portal vertex associate tegument complex; Tri1, 2A, and 2B—capsid triplex proteins; SCP—small capsid protein.

However, in the presence of compound, no processed viral pORF54 was detected in Y324C cell extracts (Figure 6B, lane 6), and virions purified from Y324C cells contained an abundance of full length pORF54^HA^ (Figure 6B, lane 10; fl v-pORF54^HA^). So, although pORF54^HA^ was incorporated into the capsid (virion) in the presence of Comp I, the reduced amount of processed product suggests that DNA encapsidation is inefficient in the presence of compounds. The results suggest the likely mechanism of action of the compound series is interference with portal structural transition (Figure 7) during the encapsidation process as opposed to preventing portal incorporation into the capsid vertex.

## 4. Discussion

Comp I belongs to a broader chemical series that includes analogs with potencies as low as ~0.03 µg/mL in vitro [35]. To contextualize these findings, we benchmark Comp I and related molecules against established VZV therapies, considering potency, mechanism of action, and resistance potential. Conventional VZV antivirals include the nucleoside analog acyclovir, the pyrophosphate analog foscarnet, the helicase–primase inhibitor amenamevir (approved in Japan), and the thymidine analog brivudine (approved in a subset of European countries).

Acyclovir requires activation by the viral thymidine kinase (TK) before incorporation by the viral DNA polymerase, resulting in chain termination. Reported EC_50_ values for VZV range from ~1–5 µg/mL [74]. Resistance primarily arises from TK gene deletions or mutations, and less frequently from DNA polymerase alterations—events mostly observed under prolonged therapy or in immunocompromised hosts [75]. Foscarnet acts as a pyrophosphate mimic, directly inhibiting viral DNA polymerase without reliance on TK activation, thereby retaining activity against TK-deficient strains. However, higher concentrations (often >25 ug/mL) are required, limiting its clinical use due to toxicity [11]. Amenamevir, a helicase–primase (HP) inhibitor acting on the ORF55/ORF6/ORF52 complex, shows EC_50_ ≈ 0.01–0.03 µg/mL, retaining activity against acyclovir-resistant and TK-negative strains [76]. Brivudine, another TK-dependent thymidine analog, demonstrates sub-µg/mL to nanomolar EC_50_ values for TK-competent VZV strains but loses activity against TK-deficient mutants, yielding a resistance profile similar to acyclovir [11].

In contrast, our compounds act through a novel mechanism—inhibition of the VZV portal protein, an essential component of the viral DNA encapsidation machinery. This protein forms the channel through which newly synthesized viral DNA is packaged into nascent capsids and whose structure is highly conserved among herpesviruses, suggesting a limited tolerance for escape mutations. Consistent with this, resistant isolates were difficult to generate in vitro, implying a high genetic barrier to resistance.

Comp I exhibits potency superior to acyclovir, and analogs in the same series achieve EC50 values near 0.03 µg/mL, approaching the best-in-class range of helicase–primase inhibitors. The combination of potential drug resistance [77,78] and vaccine hesitancy [79] make both chickenpox and shingles a continuing medical concern. We propose that α-methylbenzyl thiourea compounds merit further investigation as a treatment option for VZV infections because they act in a manner distinct from currently approved antiviral drugs. These compounds are effective against acyclovir resistant VZV strains in vitro and have been shown to target viral DNA encapsidation [33]. The studies presented here seek to better define their mechanism of action.

Viruses isolated after passage of wild type VZV in the presence of Comp I showed increased resistance, and mutations were mapped to four independent pORF54 loci: E48K, G304E, Y324C, and G407D. Based on available structural information/models, these four amino acids lie in relative proximity to each other within the tertiary structure of the portal. Employing recombineering, viruses were generated containing additional amino acid substitutions at one of the four loci. The viability of each mutant was assessed by comparing their replication phenotype to that of parental VZV_LUC_. In addition, yield reduction assays were performed to assess the ability of each mutant to replicate in the presence of increasing concentrations of Comp I compared to VZV_LUC_.

Of the twenty-one unique recombineered viruses characterized in this study, only one had a crippled replication phenotype (G407K), eight were sensitive to Comp I, and the remaining 13 acquired a resistant phenotype. Results for each locus highlighted possible requirements to retain activity or binding of methylbenzyl compounds at the portal–capsid interface (the ledge): amino acid 48 = negative charge in alerion, amino acids 304/305 = retention of loop extending into a putative pocket, and amino acid 324 = aromatic substituent necessary in the stem.

Any substitution beyond the most conservative changes at amino acids 48, 304, and 324 significantly reduced compound efficacy. Figure 7 shows details of the association of the VZV portal with B and C capsids. A white and red triangle was projected over the area containing the mutations—the portal ledge (N-terminus-wing-stem intersection) and capsid proteins. We believe these three loci contribute to, or are near, a compound binding site derived from the portal–capsid interface.

We have shown that the VZV portal dodecamer remains intact and is present in the capsid in the presence of Comp I. This suggests that Comp I may prevent either the correct positioning of the portal in the capsid vertex or prevent portal from undergoing a required conformational shift during DNA packaging, when B capsids become DNA-filled C capsids that then go on to form virions.

Whether compound binds only to the portal oligomer or fits into a pocket derived from a combination of portal and capsid proteins is not yet known. In either case, one could envision up to twelve compound binding sites around the entire circumference of the portal ledge. This could perturb the portal–capsid interface resulting in an incorrectly positioned portal incapable of proceeding through the conformational changes required to support DNA encapsidation [33,35]. As shown previously, compounds from this series are non-toxic and efficacious in vivo [34]. Their novel target (the portal–capsid interface) and activity against acyclovir resistant VZV makes them potentially important drug candidates for further development.

## Figures and Tables

**Table 1:** **Reagents and materials used in this study**.

Reagent	Description	Source, Reference or Sequence ^a^
**Bacterial Artificial Chromosomes (BACs)**		
VZV_LUC_	VZV pOKA containing firefly luciferase and green fluorescent protein	Zhang et al., 2007 [68]
D54S	galK cassette in place of ORF54 bp 301 to 1574 in VZVLUC	Visalli et al., 2014 [50]
D54L	galK cassette in place of entire ORF54 bp in VZVLUC	Visalli et al., 2014 [50]
VZV_LUC_54^HA^	VZV pOKA containing a N-terminal HA epitope tag	Visalli et al., 2024 [69]
**Plasmids**		
p*gal*K	Used to make *gal*K cassette with flanking ORF54 homology arms	Warming et al., 2005 [70]
pJET1.2	Used to clone ORF54 amplicons for sequencing	Thermo
pJET54	Used for mutagenesis of ORF54; Contains full-length ORF54 (2310) flanked by ORF53 (248 bp) and ORF55 (413 bp) sequences	Visalli et al., 2024 [69]
**Bacterial Strains**		
SW102	Used to propagate/manipulate VZV BAC clones	Warming et al., 2005 [70]
NEB 5-alpha	Competent cells for cloning	New England Biolabs
**Primers ^b^**		
MU31	Reverse partner to create amplicons with ORF55 flanks for recombineering	CGGACGACTCGCATAAGCCGTTGATAACTTA
MU23	Forward partner to create amplicons with ORF53 flanks for recombineering	CCGTATACACCCTATCTTCAACCGCAGTT
MU193	Reverse phosphorylated partner used to modify AA E48	ATATCGAACATGTTCTTGTATTGGTCATTTG
MU190	Forward phosphorylated partner used to modify AA E48D	ATACTGGAATGAcTACGCCCCGG
MU191	Forward phosphorylated partner used to modify AA E48R	ATACTGGAATagGTACGCCCCGG
MU192	Forward phosphorylated partner used to modify AA E48A	ATACTGGAATGcGTACGCCCCGG
UA634	Forward phosphorylated partner used to modify AA E48K	ATACTGGAATaAGTACGCCCCGG
MU148	Reverse phosphorylated partner used to modify AA G304	CGGCCACACGACCAAACACT
MU137	Forward phosphorylated partner used to modify AA G304E	CCCGTTGTATGTGaGGAGGGTGTAG
MU150	Forward phosphorylated partner used to modify AA G304D	CCCGTTGTATGTGacGAGGGTGTAG
MU151	Forward phosphorylated partner used to modify AA G304R	CCCGTTGTATGTaGGGAGGGTGTAG
MU149	Forward phosphorylated partner used to modify AA G304K	CCCGTTGTATGTaaGGAGGGTGTAG
MU147	Forward phosphorylated partner used to modify AA G304A	CCCGTTGTATGTGcGGAGGGTGTAG
MU198	Reverse phosphorylated partner used to modify AA Y324	ACCTCCCCAGAAAGCCGCT
MU194	Forward phosphorylated partner used to modify AA Y324F	GTTGGCCTGTTtTGCATTACGTG
MU195	Forward phosphorylated partner used to modify AA Y324W	GTTGGCCTGTTggGCATTACGTG
MU196	Forward phosphorylated partner used to modify AA Y324M	GTTGGCCTGTatgGCATTACGTG
MU210	Forward phosphorylated partner used to modify AA Y324S	GTTGGCCTGTTcTGCATTACGTG
MU209	Forward phosphorylated partner used to modify AA Y324C	GTTGGCCTGTTgTGCATTACGTG
MU197	Forward phosphorylated partner used to modify AA Y324A	GTTGGCCTGTgcTGCATTACGTG
MU233	Reverse phosphorylated partner used to modify AA G407 N408	TAGATAGGAACGTACGGTTTCG
MU222	Forward phosphorylated partner used to modify AA G407A	GAAGAAACGGcCAATCACATTCTG
MU223	Forward phosphorylated partner used to modify AA G407S	GAAGAAACGaGCAATCACATTCTG
MU224	Forward phosphorylated partner used to modify AA G407T	GAAGAAACGacCAATCACATTCTG
MU225	Forward phosphorylated partner used to modify AA G407E	GAAGAAACGGagAATCACATTCTG
MU226	Forward phosphorylated partner used to modify AA G407R	GAAGAAACGcGCAATCACATTCTG
MU227	Forward phosphorylated partner used to modify AA G407K	GAAGAAACGaagAATCACATTCTG
MU228	Forward phosphorylated partner used to modify AA N408G	GAAGAAACGGGCggTCACATTCTG
MU229	Forward phosphorylated partner used to modify AA N408A	GAAGAAACGGGCgcTCACATTCTG
MU230	Forward phosphorylated partner used to modify AA N408S	GAAGAAACGGGCAgTCACATTCTG
MU231	Forward phosphorylated partner used to modify AA N408E	GAAGAAACGGGCgAaCACATTCTG
MU232	Forward phosphorylated partner used to modify AA N408R	GAAGAAACGGGCAgaCACATTCTG
MU221	Forward phosphorylated partner used to modify AA G407D N408D	GAAGAAACGGaCgATCACATTCTG

^a^ VZV sequences: uppercase lettering; VZV point mutations: lowercase bold lettering; *gal*K sequences: lowercase lettering. ^b^ Primers are forward (F) and reverse (R) with respect to the genome map.

**Table 2:** Phenotype of BAC generated mutants.

Amino Acid Change	EC_50_ (μg/mL) *	Fold Increase	Phenotype	Substitution	Characteristics	Comments		
n/a (VZV_LUC_–OKA)	0.09 ± 0.002	n/a	S	n/a				
E48K	1.92 ± 0.15	21	R	NC	Neg > Pos	Negative charge important		
E48R	1.86 ± 0.05	21	R	NC	Neg > Pos			
E48D	0.11 ± 0.003	1.2	S	C	Neg > Neg			
E48A	1.41 ± 0.03	16	R	NC	Neg > Uncharged			
G304E	>2	22	R	NC	Uncharged > Pos	Glycine important for loop from lower wing that extends into the portal ledge		
G304K	1.31 ± 0.03	15	R	NC	Uncharged > Neg			
G304R	1.34 ± 0.06	15	R	NC	Uncharged > Neg			
G304D	0.57 ± 0.08	6	R	NC	Uncharged > Pos			
G304A	0.40 ± 0.02	4	R	C/NC	Uncharged > Pos			
Y324C	>2	22	R	NC	Aro > n/a	Less bulky aromatic important		
Y324F	0.15 ± 0.01	1.7	S	C	Aro > Aro			
Y324W	>2	22	R	C	Aro > Aro			
Y324S	>2	22	R	NC	Aro > n/a			
Y324M	>2	22	R	NC	Aro > n/a			
Y324A	>2	22	R	NC	Aro > n/a			
G407K	0.015 ± 0.001	0.2	hS	NC	Uncharged > Pos	Hypersensitivity conveyed by diverse substitutions; negative charge yields resistant phenotype		
G407R	0.021 ± 0.001	0.2	hS	NC	Uncharged > Pos			
G407D	1.56 ± 0.14	17	R	NC	Uncharged > Neg			
G407E	0.40 ± 0.02	4	R	NC	Uncharged > Neg			
G407A	0.02 ± 0.001	0.2	hS	C/NC	NP > NP			
G407T	0.015 ± 0.001	0.2	hS	NC	NP > P			
G407S	0.082 ± 0.003	0.9	S	C/NC	NP > P			

* EC_50_ values generated from yield reduction assays with Comp I. Each mutant is indicated by its amino acid substitution, and the fold increase in EC_50_ compared to VZV_LUC_. Aro = Aromatic, NP = Non-polar, P = Polar, hS = hypersensitive, R= Resistant, S = Sensitive, Neg = Negative charge, Pos = Positive charge, NC = Non-conservative, C = Conservative.

## Data Availability

All data and reagents are available upon request to the corresponding author. There were no new nucleotide or amino acids sequences.
